# Evaluation of Prostatic Lesions by Transrectal Ultrasound, Color Doppler, and the Histopathological Correlation

**DOI:** 10.7759/cureus.1422

**Published:** 2017-07-03

**Authors:** Sachin Khanduri, Gaurav Katyal, Aakshit Goyal, Saurav Bhagat, Santosh Yadav, Tarim Usmani, Nikita Singh, Mriganki Chaudhary, Shobha Khanduri

**Affiliations:** 1 Radiodiagnosis, Era's Lucknow Medical College and Hospital; 2 Radiodiagosis, Era's Lucknow Medical College and Hospital; 3 Department of Radiology, Era's Lucknow Medical College and Hospital; 4 Pathology, Era's Lucknow Medical College and Hospital

**Keywords:** transrectal ultrasound, color doppler, prostate cancer, vascularity, asymmetry

## Abstract

Objective

To evaluate the role of a transrectal ultrasound (TRUS) guided biopsy and a color Doppler test in the detection of prostate cancer in patients with increased serum prostate-specific antigen (PSA) levels and/or an abnormal digital rectal examination (DRE).

Method

A total of 40 cases, ranging in age from 50 to 80 years and showing increased serum PSA levels (4-10 ng/ml) and/or abnormal DRE, were enrolled and underwent TRUS evaluation followed by color Doppler flowmetric studies. A TRUS-guided biopsy was performed in all the cases. The findings were confirmed histopathologically. Data were analyzed using the chi-square test.

Results

Histopathologically, a total of 13 cases (32.5%) were malignant. On TRUS, irregular shape, heterogeneous echotexture loss of differentiation between the peripheral and internal zones, less mean weight of the prostate, and capsular invasion were significantly associated with malignancy. On flowmetry, moderate vascularity and focal asymmetry were significantly associated with malignancy. The combined use of TRUS and color Doppler flowmetry was found to be 100% sensitive and 92.6% specific and had a positive predictive value (PPV) and a negative predictive value (NPV) of 86.7% and 100%, respectively.

Conclusion

TRUS with color Doppler flowmetry was highly sensitive and specific in the detection of prostate malignancy.

## Introduction

Prostate cancer is the most common malignancy in men in the United States, with approximately 192,280 cases diagnosed yearly [1]. Globally too, prostate cancer happens to be the second-most common cancer among males, with annual incidence reaching up to 679,060 cases [2]. About 0.19 million new cases of this disease are projected for men in the United States and 25,000 for men in Canada, with a mortality rate nearing 25% in both countries [2]. In India, at an all-India level, projected cases for prostate cancer for the periods 2010, 2015, and 2020 were estimated at 26,120, 28,079, and 30,185, respectively [3], with 5-year mortality rates being as high as 36% [4]. Age adjustments based on incidence rates for prostate cancer have shown an increase, ranging from 20% to 100% in five major cancer registries of India (Mumbai, Bangalore, Chennai, Delhi, and Bhopal) in a period of 20 years, starting from 1982 to 2002 [5].

The diagnosis and treatment of prostate cancer are very challenging. The current methods of screening for prostate cancer include measuring serum prostate specific antigen levels (PSA), digital rectal examinations (DREs), and transrectal ultrasound (TRUS). Scanning and biopsy confirm the diagnosis; however, the sensitivity and specificity of TRUS for diagnosing prostate cancer still needs further study. A color Doppler ultrasound, because of its ability to effectively visualize vascular changes, provides a better diagnostic as well as prognostic value. Prostate cancer, in common with many other tumors, displays increased angiogenesis, resulting in increased microvessel density [6-7]. Tumor blood vessels also have random pathways and increased tortuosity; these changes could help for easy tumor detection through a color or power Doppler examination [6]. Increased color Doppler blood flow tends to indicate more aggressive tumors with higher Gleason grades as well as a higher risk of recurrence [8-9]. Despite these promising findings, some workers have highlighted doubts over the efficiency of color Doppler flowmetry because of resolution issues, consequently limiting the detection of hypervascular nodules [10]. However, to counter this, some studies highly advocate the systematic use of color Doppler ultrasounds during targeted biopsies because of the tendency to diagnose higher grade and significant cancers. In recent years, the use of high-resolution color doppler ultrasounds (HR-CDUs) and tissue harmonic technology has improved cancer detection. In addition, a specific lesion-directed target biopsy along with a biopsy of the potential route of tumor escape (such as a nearby neurovascular bundle and seminal vesicles) improved the staging of the cancer and often improved Gleason grading [11]. Considering these advantages of a color doppler test, it is gaining popularity as a diagnostic modality for differentiating between various prostatic lesions with a reported benefit over the conventionally used TRUS approach.

In view of the promising role of a color doppler ultrasound in the diagnosis of prostate cancer, the present study was undertaken to evaluate the role of color doppler guided TRUS with TRUS imaging in the evaluation of suspected cases of prostate malignancy.

## Materials and methods

The study was carried out on a total of 40 male patients, aged 50-80 years, with serum PSA levels of 4-10 ng/ml in the absence of urinary tract infections, acute urinary retention, acute prostatitis, or recent catheterization and having a hard, enlarged nodular prostate on DRE. The project was approved by the Institutional Ethical Committee. Informed consent was obtained from all the participants. All suspected patients attending the surgical outpatient/inpatient of our institution who fulfilled the inclusion criteria were examined in the left lateral decubitus, knee-chest position and were subjected to DRE followed by TRUS with a color doppler for the detection of prostatic lesion using G.E. LOGIQ 5 PRO (GE Healthcare, Little Chalfont, United Kingdom) Ultrasonography (USG) color doppler machine (with a TRUS probe (6-10 MHz)). Later, a TRUS guided biopsy was performed using an 18G biopsy gun to confirm the radiological diagnosis.

On TRUS, the number of nodules, zone involved, size and shape of the lesion, echogenicity, echotexture, difference between peripheral and internal zone, prostate weight, and capsular invasion were noted. Doppler color flowmetry studies were done for the extent of vascularity (mild/moderate) and vascular asymmetry (focal or diffuse).

Data Analysis

Data were analyzed using Statistical Package for Social Sciences (SPSS) version 21.0. A Chi-square test and a ‘t’-test of independent samples were used to compare the data. A ‘p’ value less than 0.05 indicated a significant association. Diagnostic efficacy was expressed in terms of sensitivity, specificity, positive predictive value (PPV), negative predictive value (NPV), and accuracy.

## Results

The age of patients ranged from 51 to 77 years. The mean age of patients was 63.80±6.76 years. A majority of the patients were less than 65 years of age (65%). On DRE, a total of 17 patients (42.5%) had induration while 23 (57.5%) had nodular lesions. PSA values ranged from 5.8 to 9.8 ng/ml. Exactly half the patients had PSA less than 8 ng/ml. Histopathologically, 13 cases (32.5%) were malignant. On TRUS evaluation, a total of 10 cases (25%) were malignant. TRUS findings combined with color Doppler vascularity findings diagnosed malignancy in 15 cases (37.5%) (Table 1).

**Table 1:** The general and clinical profiles of patients TRUS, color Doppler, and histopathological diagnosis of these patients are also indicated. Digital rectal examination (DRE) Prostate-specific antigen (PSA) Transrectal ultrasound (TRUS)

SN	Characteristics	Statistics
1.	Mean age ± SD (range) in years 63.80 ± 6.76 (51-77)	
	<65 yrs	26 (65.0%)
	>65 yrs	14 (35.0%)
2.	DRE findings	
	Induration	17 (42.5%)
	Nodule	23 (57.5%)
3.	Mean PSA ± SD (range) (ng/ml) 7.96 ± 1.09 (5.8-9.8)	
	<8	20 (50.0%)
	>8	20 (50.0%)
4.	Histopathological diagnosis	
	Benign	27 (67.5%)
	Malignant	13 (32.5%)
5.	TRUS (grey scale diagnosis)	
	Benign	30 (75.0%)
	Malignant	10 (25.0%)
6.	TRUS + color doppler	
	Benign	25 (62.5%)
	Malignant	15 (37.5%)

On comparing TRUS and color Doppler flowmetric findings between histopathologically benign and malignant cases, irregular shape, heterogeneous echotexture, loss of differentiation between peripheral and internal zones, less mean prostate weight, and capsular invasion were found to be significantly associated with malignancy (p<0.05). On color Doppler assessment, moderate vascularity and focal vascular asymmetry were found to be significantly associated with malignancy.

**Figure 1:**
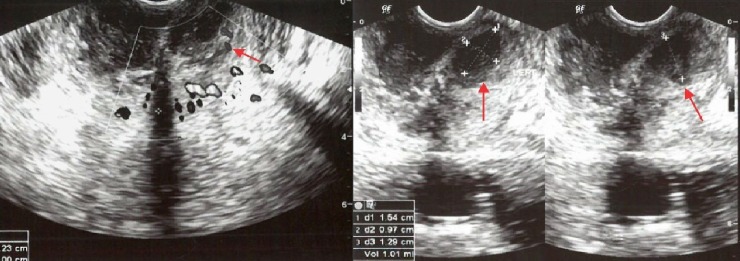
TRUS and power Doppler in a 46-year-old male The image shows multiple irregular hypoechoic nodules (arrow). Color and power Doppler demonsrate increased vascularity. Biopsy revealed inflammatory changes.

**Figure 2:**
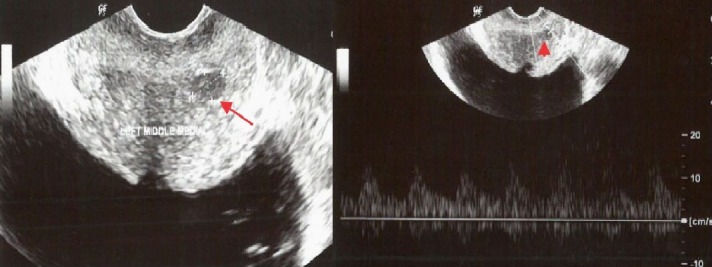
TRUS and power Doppler in a 54-year-old male The images show a small, well-defined, oval-shaped hypoechoic nodule (arrow) in the peripheral zone. The color Doppler image shows focal increased vascularity in the region of the nodule. DRE was negative and PSA was slightly increased.

**Figure 3:**
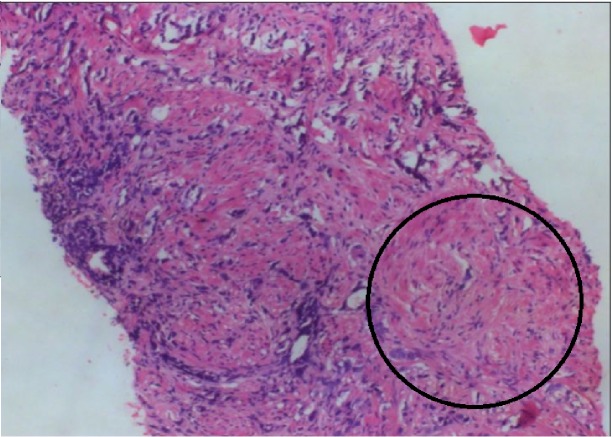
Histopathological section of prostate in the same 54-year-old patient The image shows a variable-sized infiltrating gland with a tiny cluster of cells in hypernephroid pattern. Hyperchromatic nuclei of cells lining the glands are also seen, suggesting malignancy (black arrow).

On evaluating the diagnostic efficacy of the combination of TRUS diagnosis with color Doppler flowmetry for vascular asymmetry (focal) against histopathological diagnosis for malignancy, a total of 13 cases were found to be true positive, 2 were false positive, none were false negative, and 25 were true negative. Correspondingly, the sensitivity, specificity, positive predictive, negative predictive, and accuracy values were 100%, 92.6%, 86.7%, 100%, and 95% respectively (Figures 1-3).

## Discussion

Recent advances in sonographic vascular imaging, such as color flow Doppler (CFD) and power Doppler imaging (PDI), have given rise to new hope for better prostate cancer diagnosis. It has been well-documented that cancers have neovascularity and can incite a vascular response. This has been reported with a variety of cancers as well as with prostate cancer [12-14]. In the present study, histopathological examination revealed a malignancy rate of 32.50%. The malignancy rate has shown considerable variability from 29.0% to 53% in different case series using different imaging modalities for the evaluation of prostate cancer [15-21]. Most of these case series had a PSA/DRE screened population as in the present study The malignancy rates in the present study are comparable to those reported by Shigeno et al. (31.3%), Halpern et al. (29.0%), and Remzi et al. (34.7%) [15-17]. 

In the present study, TRUS diagnosis established 30 cases (75%) as benign and 10 cases (25%) as malignant, showing the rate of cancer detection to be close to that diagnosed through histopathology. Among different TRUS characteristics, irregular shape, heterogeneous echotexture, loss of differentiation between the peripheral and internal zones, increased mean prostate weight, and capsular invasion were found to be significantly associated with malignancy. Ismail et al. (2001) [19] reported capsular invasion to be associated with the advancing stage of the disease. Novis et al. (2006) [20], on the other hand, also reported extracapsular extension as the distinguishing feature for malignancy (Table 2). 

**Table 2:** A comparison of TRUS and color Doppler findings between malignant and benign cases The comparison has been made based on various characteristics of the nodules.

S. No.	Finding	Total (N=40)			Histopathological diagnosis			Statistical significance	
				Benign (n=27)		Malignant (n=13)			
		No.	%	No.	%	No.	%	c^2^	p
1-	Number of nodules								
	1	18	45	12	44.44	6	46.15	0.195	0.907
	2	8	20	5	18.52	3	23.08		
	>2	14	35	10	37.04	4	30.77		
2-	Zone involved								
	Apex	3	7.5	3	11.11	0	0	1.751	0.626
	Base	10	25	7	25.93	3	23.08		
	U/L peripheral	22	55	14	51.85	8	61.54		
	U/L transitional	5	12.5	3	11.11	2	15.38		
3-	Size								
	≤2 cm^2^	2	5	1	3.7	1	7.69	1.659	0.436
	2-5 cm^2^	18	45	14	51.85	4	30.77		
	>5 cm^2^	20	50	12	44.44	8	61.54		
4-	Shape								
	Irregular	12	30	2	7.41	10	76.92	20.955	<0.001
	Oval	11	27.5	9	33.33	2	15.38		
	Round	9	22.5	9	33.33	0	0		
	Round to oval	8	20	7	25.93	1	7.69		
5-	Echogenicity								
	Hypoechoic	22	55	12	44.44	10	76.92	3.874	0.144
	Iso to hypoech.	17	42.5	14	51.85	3	23.08		
	Isoechoic	1	2.5	1	3.7	0	0		
6-	Echotexture								
	Heterogeneous	11	27.5	1	3.7	10	76.92	23.595	<0.001
	Homogenous	29	72.5	26	96.3	3	23.08		
7-	Difference between peripheral								
	and internal zones								
	Lost	10	25	0	0	10	76.92	27.692	<0.001
	Maintained	30	75	27	100	3	23.08		
8-	Prostate weight (gm)	35.08+7.95		38.32+8.41		32.14+6.36		‘t’=2.633; p=0.012	
		(25-56)			(28-56)			(25-52)	
	21-30 gm	15	37.5	9	33.33	6	46.15	2.349	0.309
	31-40 gm	16	40	13	48.15	3	23.08		
	>40 gm	9	22.5	5	18.52	4	30.77		
9-	Capsular invasion	4	10	0	0	4	30.77	9.231	0.002
10-	Moderate vascularity	20	50	9	33.33	11	84.62	9.231	0.002
11-	Focal vascular	13	32.5	2	7.41	11	84.62	23.84	<0.001
	asymmetry								

In the present study, during a Doppler assessment, we focused on only two characteristics: The first was the presence of vascularity and the second was the asymmetry of vascularity. Both these findings were found to be significantly associated with malignancy. Vascularity and its intensity and pattern are the Doppler characteristics that are generally focused on for malignancy detection, and almost all studies focus on this characteristic [17,20-21,23]. In the present study, we focused on moderate and focal hypervascularity as the diagnostic criteria for color doppler. A combination of TRUS and Doppler vascular differentiation achieved high sensitivity (100%) as well as specificity (92.6%) with an excellent NPV (100%) and a good PPV (86.7%) (Table 3).

**Table 3:** The results of the diagnostic efficacy of TRUS with color Doppler against histopathology The statistical results of the study Positive predictive value (PPV); negative predictive value (NPV)

		Diagnosis		
TRUS + color	Histopathological diagnosis			Total
Doppler diagnosis				
	Malignant		Benign	
Malignant	13 (TP)		2 (FP)	15
Benign	0 (FN)		25 (TN)	25
Total	13		27	40
Sensitivity	Specificity	PPV	NPV	Accuracy
100%	92.6%	86.7%	100.0%	95.0%

The combination of TRUS and color Doppler has resulted in varying diagnostic efficiency in different studies. Generally, the sensitivity of the combined evaluation has been shown to lie in the range 33% [22] to 88.23% [23] and specificity has been shown to lie between 57% [23] and 85% [23-24]. Drudi et al. (2006) [24], in their study, set their eyes on selecting more-specific criteria and could achieve poor sensitivity (38.5%) but high specificity (85%). The combinatorial criteria using “both positive” end up with high specificity but low sensitivity. In the present study, had we used these criteria, we would also have ended up achieving sensitivity less than 52.4% but could have achieved a specificity of 100%. This was not intended, as we already had an absolute (100%) specificity for TRUS and there was no scope for increasing the specificity further.

However, those researchers who skillfully focused on the use of both the modalities for maximizing the sensitivity/specificity combination could achieve high sensitivity as well as specificity, as with Sen et al. (2008) [23]. They, before combinatorial use, had high rates of sensitivity (73.5% and 88.23%) for both TRUS as well as doppler, but had poor specificity for both modalities (33.3% and 66.7%); they focused on the approach of selecting the ‘both positive’ approach for selected Doppler and TRUS characteristics and improved their specificity rate to 73.52%. In the present study, we wanted to increase sensitivity at the cost of specificity and could achieve absolute sensitivity (100%) while maintaining excellent specificity (92.6%). The findings of the present study were encouraging from the point of view of using a combinatorial approach to achieve higher sensitivity as well as specificity and, consequently, overcoming the shortcomings of the independent use of TRUS as well as color doppler. 

However, this dual approach could be a practical problem in view of the discomfort caused to patients and the practical use of this approach in clinical settings being subject to the unavailability of a better and single modality. Fortunately, modalities such as contrast-enhanced magnetic resonance imaging (MRI) imaging are available, which have shown to be better than both these modalities [22] and, as a result, wherever these advanced modalities are available, the use of dual assessment is not recommended. However, considering the fact that facilities such as contrast-enhanced MRI are not available at all centers and, therefore, in low-resource settings, the combinatorial approach of TRUS with color Doppler could be recommended for the assessment of suspicious prostate cancer. In addition, because the criteria for combinatorial use have been shown to change in different studies, further studies to potentiate which combination of criteria will provide the optimum result are recommended.

## Conclusions

The findings of the present study showed that TRUS with color Doppler flowmetry can play an important role in the detection of prostate malignancy, with high sensitivity as well as specificity. The high NPV, as observed in the present study, could avoid unnecessary diagnostic invasive intervention. However, given the limitation of the sample size, the findings in the present study should be used cautiously with further corroboration by a larger sample size.

## References

[1] Jemal A, Siegel R, Ward E (2009). Cancer statistics, 2009. CA Cancer J.

[2] Ma X, Yu H (2006). Global burden of cancer. Yale J Biol Med.

[3] Lalitha K, Suman G, Pruthvish S (2012). Estimation of time trends of incidence of prostate cancer-an Indian scenario. Asian Pac J Cancer Prev.

[4] Balasubramaniam G, Talote S, Mahantshetty U (2013). A hospital-based survival study from Mumbai, India. Asian Pac J Cancer Prev.

[5] Yeole BB (2008). Trends in the prostate cancer incidence in India. Asian Pac J Cancer Prev.

[6] Kay PA, Robb RA, Bostwick DG (1998). Prostate cancer microvessels: a novel method for three-dimensional reconstruction and analysis. Wiley Online Library.

[7] Louvar E, Littrup PJ, Goldstein A (1998). Correlation of color doppler flow in the prostate with tissue microvascularity. Wiley Online Library.

[8] Cheng S, Rifkin MD (2001). Color doppler imaging of the prostate: important adjunct to endorectal ultrasound of the prostate in the diagnosis of prostate cancer. Ultrasound Q.

[9] Huang ST, Hsieh ML (2008). Evaluation of resistance index in patients with prostate cancer. Anticancer Res.

[10] Arger P, Malkowicz S, VanArsdalen K (2004). Color and power doppler sonography in the diagnosis of prostate cancer. J Ultrasound Med.

[11] Lee F, Bahn DK, Siders DB (1998). The role of TRUS-guided biopsies for determination of internal and external spread of prostate cancer. Semin Urol Oncol.

[12] Brawer MK, Deering RE, Brown M (1994). Predictors of pathologic stage in prostatic carcinoma: the role of neovascularity. Cancer.

[13] Gasparini G, Harris AL (1999). Prognostic significance of tumor vascularity. Springer Link.

[14] Evans SM, Laughlin KM, Pugh CR (1997). Use of power doppler ultrasound to locate regions of tumor hypoxia. Br J Cancer.

[15] Shigeno K, Igawa M, Shiina H (2000). The role of colour doppler ultrasonography in detecting prostate cancer. BJU Int.

[16] Halpern EJ, Frauscher F, Strup SE (2002). High-frequency doppler US of the prostate: effect of patient position. Radiology.

[17] Remzi M, Dobrovits M, Reissigl A (2004). Can power doppler enhanced transrectal ultrasound guided biopsy improve prostate cancer detection on first and repeat prostate biopsy?. ESOU.

[18] Inahara M, Suzuki H, Nakamachi H (2004). Clinical evaluation of transrectal power doppler imaging in the detection of prostate cancer. Int Urol Nephrol.

[19] Ismail M, Gomella LG (2001). Ultrasound for prostate imaging and biopsy. Curr Opin Urol.

[20] Novis M, BaroniI R, CerriI L (2011). Clinically low-risk prostate cancer: evaluation with transrectal doppler ultrasound and functional magnetic resonance imaging. Clinics.

[21] Del Rosso A, Di Pierro ED, Masciovecchio S (2012). Does transrectal color doppler ultrasound improve the diagnosis of prostate cancer?. Arch Ital Urol Androl.

[22] Beyersdorff D, Taupitz M, Winkelmann B (2002). Patients with a history of elevated prostate-specific antigen levels and negative transrectal US-guided quadrant or sextant biopsy results: value of MR imaging. Radiology.

[23] Sen J, Choudhary L, Marwah S (2008). Role of color doppler imaging in detecting prostate cancer. Asian J Surg.

[24] Drudi FM, Giovagnorio F, Carbone A (2006). Doppler contrast sonography in the diagnosis of local recurrence after radical prostatectomy – comparison with MRI. Ultraschall Med.

